# Emotion Regulation and Brain Mechanisms: A Transdiagnostic Perspective on Neural Circuits, Individual Differences, and Clinical Translation

**DOI:** 10.3390/bs16101800

**Published:** 2026-10-02

**Authors:** Sandra Carvalho

**Affiliations:** Psychology Research Centre (CIPsi), Department of Basic Psychology, School of Psychology, University of Minho, 4710-057 Braga, Portugal; sandrarc@psi.uminho.pt

**Keywords:** emotion regulation, transdiagnostic psychopathology, prefrontal cortex, amygdala, cognitive reappraisal, neuromodulation, early-life adversity, precision psychiatry

## Abstract

Emotion regulation (ER) difficulties are now firmly established as a transdiagnostic feature of psychiatric and stress-related disorders. Large-scale meta-analyses published in 2026 confirmed ER impairments across fifteen diagnostic categories, shifting the key question from whether ER is transdiagnostic to how this knowledge can inform diagnosis and treatment. This Perspective argues that ER should be conceptualized as a transdiagnostic neurocognitive dimension rather than a disorder-specific deficit. To support this view, three complementary studies are integrated: neuroimaging studies of prefrontal-limbic and large-scale brain networks involved in cognitive reappraisal; evidence of ER-related neural alterations across mood, anxiety, trauma-related, personality, and addictive disorders; and research on individual differences and neuromodulation that explain variability in regulatory capacity and treatment response. ER is framed within a broader executive-function model, operating as a “hot” executive process alongside “cool” cognitive-control functions. Building on this evidence, a Layered Transdiagnostic Model of Emotion Regulation (LTM-ER) is proposed. The model includes a shared transdiagnostic core circuit (Layer 1), disorder-specific neural signatures (Layer 2), and individual moderators such as developmental history, dispositional traits, regulatory strategies, and stimulation parameters (Layer 3). Five testable predictions are derived from the model, highlighting how multimodal biomarkers and personalized interventions may advance precision approaches to emotion dysregulation across psychiatric disorders.

## 1. Introduction

Emotion dysregulation is not the property of any single psychiatric category. It appears, in varying forms, in mood disorders, anxiety disorders, post-traumatic stress disorder, personality disorders, substance use disorders, and stress-related conditions following early adversity, and it has increasingly been proposed as a core transdiagnostic process that cuts across the categorical boundaries of current diagnostic systems (Blay et al., 2024; Morawetz et al., 2025). Over the past three years, this transdiagnostic reframing has been reinforced by two parallel developments. First, neuroimaging meta-analyses have begun to formally test, rather than merely assume, whether emotion regulation (ER) has a neural signature that is distinct from other Research Domain Criteria (RDoC) constructs and that recurs across clinical populations independently of diagnosis (Morawetz et al., 2025). Second, the field of ER research itself has been called to broaden its methodological scope, moving beyond single-strategy, single-timepoint laboratory paradigms toward more ecologically valid, multimodal, and individual-difference-sensitive designs (Petrova & Gross, 2023).

Two recent large-scale meta-analyses provide substantial additional support for the transdiagnostic relevance of ER difficulties. A systematic review and meta-analysis of 188 studies, encompassing 563 case–control comparisons across 15 psychiatric diagnoses and more than 20,000 participants, found large and significant deficits on the Difficulties in Emotion Regulation Scale in every one of the twelve disorders for which this measure was analyzed, with effect sizes ranging from 0.94 in bipolar disorder to 2.55 in borderline personality disorder, and reduced cognitive reappraisal together with elevated expressive suppression in nearly every diagnostic category examined (Sloan et al., 2026). An independent multilevel meta-analysis of 619 studies spanning almost three decades, comprising over 78,000 clinical and control participants, reached the same conclusion from a different methodological angle, reporting a large overall standardized mean difference in self-reported ER difficulties between clinical and nonclinical samples, alongside disorder-specific gradients in which some facets, including difficulties in acceptance in personality disorders and rumination together with reduced reappraisal in depressive disorders, are more pronounced than others (Clamor et al., 2026).

Taken together, these syntheses strongly support the transdiagnostic relevance of self-reported ER difficulties. Nevertheless, differences across diagnoses, measures, and study designs remain important when interpreting the extent and nature of this convergence. The present Perspective therefore focuses on the mechanistic and translational questions arising from this evidence. Specifically, the questions addressed here are: which convergent and divergent brain circuits plausibly generate this cross-diagnostic phenotype, which individual-difference variables determine why comparable adversity or comparable diagnoses produce divergent regulatory trajectories and divergent treatment responses, and how mechanistic interventions such as neuromodulation can be designed, targeted, and reported so that they test, rather than merely assume, engagement of a shared regulatory circuit. Addressing these three questions together, rather than as separate studies, is the specific contribution this article seeks to make, and it is what distinguishes this Perspective from prior transdiagnostic and meta-analytic treatments of the topic.

This Perspective takes up that agenda and asks a more applied question: what do recent brain-mechanism findings tell us about why ER fails in some individuals and some contexts, and what does this mean for designing interventions that generalize across diagnoses rather than being retrofitted to single disorders? I approach this question from a neurocognitive framework in which executive dysfunction, spanning both “cool” processes such as working memory and inhibitory control and “hot” processes such as ER and motivational control, is treated as a shared vulnerability across psychiatric conditions and an intermediate phenotype linking genetic liability, distributed circuit disruption, and everyday functional impairment (Carvalho, 2026; Carvalho et al., 2026). Within that framework, ER is not a peripheral add-on but one of the clearest examples of a hot executive process whose neural implementation can be probed directly, including through non-invasive brain stimulation, and whose individual variability has direct clinical consequences. I also draw on findings from early-life trauma research, including data from a Portuguese cohort, to illustrate how personality, coping, and attachment-related individual differences shape the translation from adverse experience to adult emotional dysregulation, differences that plausibly have a neural basis and that are rarely incorporated into mechanistic ER models.

A further motivation for this synthesis is practical rather than purely theoretical. Diagnostic manuals continue to organize treatment guidelines around categorical labels, yet clinicians routinely observe that the same regulatory difficulties, difficulty disengaging from negative affect, reliance on suppression rather than reappraisal, and heightened reactivity to interpersonal triggers, recur across patients who would never share a single diagnostic code. If ER genuinely has a partly shared neural implementation across these presentations, then treatments that target the underlying regulatory mechanism, rather than the surface diagnostic category, should in principle generalize more broadly and could be deployed earlier, before a categorical diagnosis has even been established. Evaluating that possibility requires bringing together four studies that are usually reviewed separately: the circuit-level neuroimaging literature on reappraisal and related regulatory strategies, a disorder-by-disorder account of how ER impairment manifests neurally across diagnostic categories, the clinical and epidemiological literature on how early adversity and personality shape adult regulatory capacity, and the interventional literature on non-invasive brain stimulation as a tool for modulating regulatory circuits directly. This Perspective attempts that integration, organized around the argument that individual differences, in circuitry, in developmental history, and in response to stimulation parameters, are not noise to be averaged away in this literature, but the central explanatory variable that any transdiagnostic, mechanism-based model of ER must accommodate.

Stated explicitly, this Perspective adds four things to the existing literature. First, Section 3 provides a disorder-by-disorder synthesis organized around the relationship between shared and diagnosis-associated neural findings, drawing on behavioral and neuroimaging meta-analyses (Clamor et al., 2026; Sloan et al., 2026; Morawetz et al., 2025; Wong et al., 2026). First, Section 3 provides a disorder-by-disorder synthesis organized around the relationship between shared and diagnosis-associated neural findings, drawing on behavioral and neuroimaging meta-analyses (Clamor et al., 2026; Sloan et al., 2026; Morawetz et al., 2025; Wong et al., 2026).

**Figure 1:**
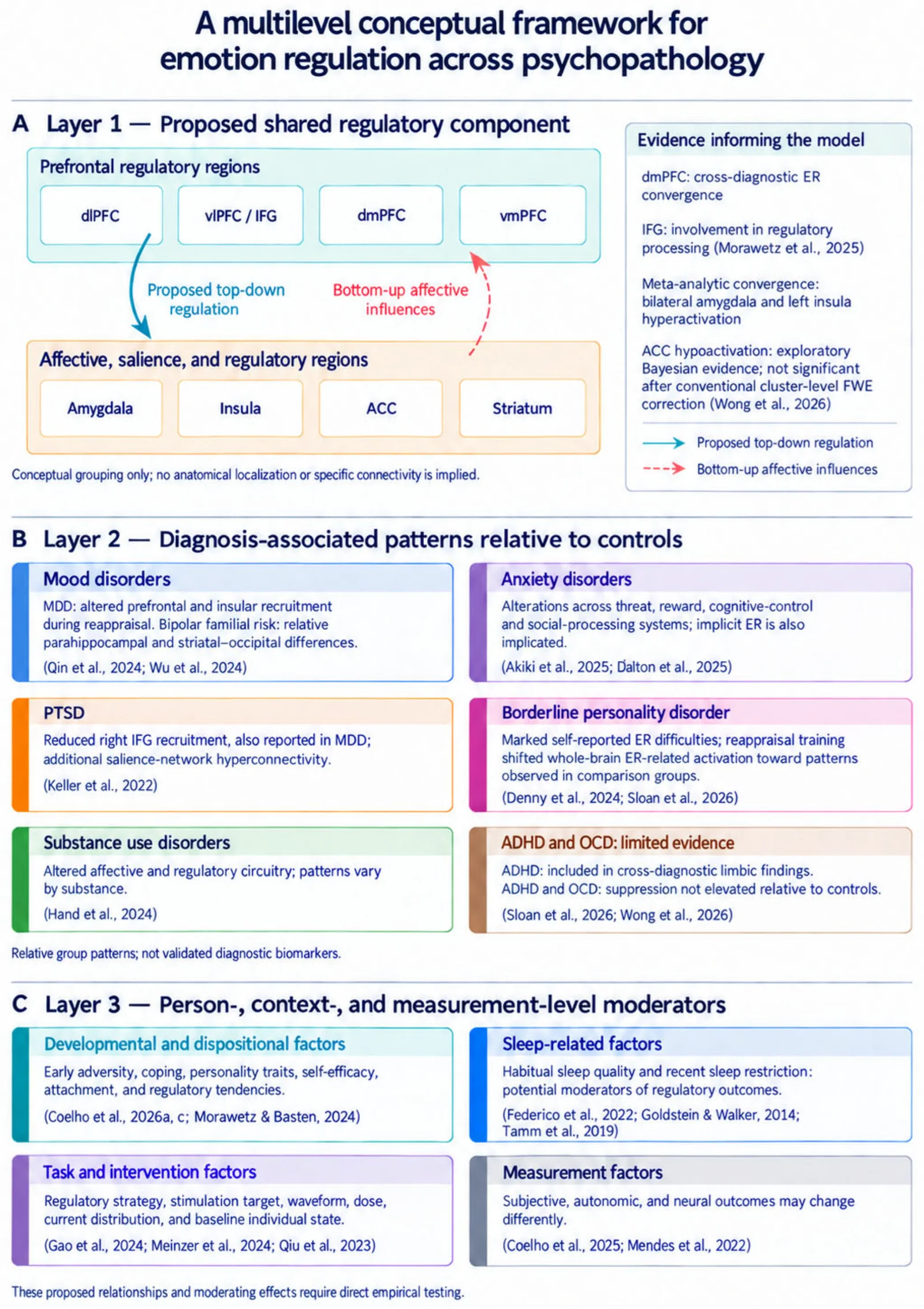
The Layered Transdiagnostic Model of Emotion Regulation (LTM-ER). (**A**) Proposed shared regulatory component, showing conceptually grouped prefrontal regulatory regions and affective, salience, and regulatory regions. Solid teal arrows indicate proposed top-down regulatory influences from prefrontal to affective and regulatory systems, whereas dashed red arrows indicate bottom-up affective influences on prefrontal regulatory processes. The schematic does not imply anatomical localisation or specific structural or functional connectivity. Findings are distinguished by evidential status (Morawetz et al., 2025; Wong et al., 2026). (**B**) Diagnosis-associated patterns relative to controls. These relative group patterns should not be interpreted as validated diagnostic biomarkers (Wu et al., 2024; Qin et al., 2024; Akiki et al., 2025; Dalton et al., 2025; Keller et al., 2022; Sloan et al., 2026; Denny et al., 2024; Hand et al., 2024; Wong et al., 2026). (**C**) Person-, context-, and measurement-level moderators, including developmental and dispositional characteristics, sleep-related variation, task and intervention factors, and measurement factors. Proposed relationships and moderating effects require direct empirical testing (Coelho et al., 2026a, 2026c; Morawetz & Basten, 2024; Goldstein & Walker, 2014; Federico et al., 2022; Tamm et al., 2019; Gao et al., 2024; Qiu et al., 2023; Meinzer et al., 2024; Coelho et al., 2025; Mendes et al., 2022).

Second, it formalizes the resulting pattern as a three-layer theoretical model, the Layered Transdiagnostic Model of Emotion Regulation (LTM-ER; Section 6 and Figure 1), that distinguishes a shared core circuit, disorder-specific circuit fingerprints, and individual-difference moderators, and that generates testable predictions rather than a descriptive summary. Third, it positions ER within a broader neurocognitive account of executive dysfunction as a transdiagnostic dimension (Carvalho, 2026; Carvalho et al., 2026) and brings early-adversity mediation findings, including from a Portuguese cohort (Coelho et al., 2026a, 2026b), into a mechanistic ER model from which such individual-difference data are usually absent. Fourth, it translates the model into concrete, actionable guidance for research design, the specification and reporting of neuromodulation trials, and clinical assessment, collected in Section 8, so that the transdiagnostic status of ER can begin to shape practice rather than remaining a conclusion drawn only in meta-analyses.

This Perspective presents a selective narrative synthesis rather than a systematic review, focusing on psychological and neuroscientific mechanisms of ER. Its organizing focus is the relationship between cross-diagnostic ER difficulties, neural findings, individual differences, and neuromodulation. The synthesis draws on systematic reviews and meta-analyses to contextualize broader patterns, alongside selected primary studies illustrating mechanistic questions, heterogeneity, and null findings. The evidence is considered in relation to the construct measured, population, task, and study design. The resulting framework is intended to generate testable hypotheses; it does not provide an exhaustive account of the literature or a formal assessment of the certainty of evidence.

## 2. Neural Circuits of Emotion Regulation: From Prefrontal-Limbic Models to Network-Level Accounts

The dominant model of ER, particularly for cognitive reappraisal, has long emphasized top-down modulation of subcortical affective structures, chiefly the amygdala, by lateral and medial prefrontal regions. That architecture was consolidated by a first generation of synthetic reviews and meta-analyses of reappraisal neuroimaging, which converged on dorsolateral, ventrolateral, and dorsomedial prefrontal cortex, dorsal anterior cingulate cortex, and posterior parietal cortex as the regulatory set that modulates amygdala responding (Buhle et al., 2014; Etkin et al., 2015; Ochsner et al., 2012). Combined transcranial magnetic stimulation (TMS) and functional magnetic resonance imaging (fMRI) work published in the last three years has provided some of the clearest causal evidence for this architecture to date. In a study combining repetitive TMS over the right ventrolateral prefrontal cortex (VLPFC) with an emotion-regulation fMRI task, facilitating VLPFC activity increased coupling with the ventromedial prefrontal cortex (VMPFC) and reduced amygdala and insula activity specifically during reappraisal, but not during passive viewing, supporting an indirect pathway in which the VMPFC serves as a relay between the VLPFC and subcortical affective regions (He et al., 2023). This causal, circuit-level evidence complements a growing body of correlational work showing that individual differences in both self-reported regulation success and ER-related traits map onto activity in the lateral prefrontal cortex, while amygdala reactivity relates more specifically to habitual regulatory tendencies (Morawetz & Basten, 2024). Resting-state effective-connectivity analyses converge on the same conclusion from a different angle: connectivity within reappraisal-related networks at rest, independent of any regulation task, predicts how successfully individuals subsequently downregulate high- and low-intensity negative emotion, suggesting that regulatory capacity is at least partly a stable property of intrinsic network organization rather than a purely task-evoked phenomenon (Morawetz et al., 2024).

A second, related shift in the last few years has been the move from a strictly prefrontal-amygdala account toward a network-level framing in which large-scale systems, including the default mode network (DMN), are implicated in emotion–cognition interactions relevant to mental health. Reviews of DMN development and function highlight its role in self-referential processing and its documented dysregulation across mood, anxiety, and stress-related conditions, situating classical reappraisal circuits within a broader landscape of intrinsic connectivity patterns that shape emotional life from childhood through adulthood (Azarias et al., 2025). This network-level view does not replace the prefrontal-limbic model; it contextualizes it, clarifying that reappraisal-related prefrontal-subcortical coupling operates within, and is constrained by, broader patterns of large-scale connectivity that also differ across individuals and across diagnostic groups.

The neural architecture emphasized here is supported most directly by studies of cognitive reappraisal. Although other regulatory processes are considered, the same pattern of regional recruitment and connectivity should not be assumed to apply uniformly to acceptance, distraction, expressive suppression, situation selection, or other ER strategies. Whether the proposed shared and divergent components generalize across strategies is an empirical question.

Figure 1 makes this article’s central hypothesis, formalized in Section 6 as the Layered Transdiagnostic Model of Emotion Regulation (LTM-ER), explicit in visual form: emotion-regulation circuitry is organized around a shared, transdiagnostic core (Layer 1) that is overlaid by disorder-specific divergence (Layer 2) and modulated by individual-difference moderators (Layer 3), rather than being disorder-bound from the outset. Panel A summarizes the prefrontal-limbic circuitry schematically, distinguishing two independent, empirically derived transdiagnostic convergence signatures from the broader set of prefrontal and limbic regions that support top-down regulation and bottom-up affective signaling, and marking the two prefrontal targets most frequently used in the neuromodulation studies reviewed in Section 5. Panel B links six diagnostic categories to that same core, each annotated with its disorder-specific circuit fingerprint as reviewed in Section 3. Panel C lists the four families of individual-difference moderators, reviewed in Section 4 and Section 5, that determine how strongly the first two layers are expressed in a given person and how that person responds to intervention. The figure is thus the visual equivalent of this Perspective’s argument that ER dysfunction reflects a shared core deficit overlaid by disorder-specific network alterations and shaped by individual history and disposition, rather than six unrelated phenomena.

(A) Layer 1. Teal arrows denote the top-down regulatory pathway by which lateral and medial prefrontal regions (dlPFC, VLPFC, VMPFC) modulate limbic and salience-related structures (amygdala, insula, anterior cingulate cortex [ACC], striatum) during cognitive reappraisal (He et al., 2023; Morawetz & Basten, 2024; Morawetz et al., 2024). Dashed rust arrows denote bottom-up affective signaling from limbic structures back to the prefrontal cortex. The dark teal node (dmPFC/IFG) marks the region whose engagement during ER recurs most consistently across diagnostically heterogeneous clinical samples in coordinate-based meta-analyses and is treated here as one transdiagnostic convergence signature (Morawetz et al., 2025). The dashed gold outline marks a second, independently derived transdiagnostic core, the amygdala/insula hyperactivation and ACC hypoactivation pattern identified by activation-likelihood-estimation meta-analysis across borderline personality disorder, ADHD, and bipolar disorder (Wong et al., 2026). Gold stars mark the dlPFC and VMPFC as the two prefrontal targets most frequently probed with non-invasive brain stimulation in the studies reviewed in Section 5 (Coelho et al., 2025; Gao et al., 2024).
(B) Layer 2. Six diagnostic categories, each linked by a leader line to the transdiagnostic core, annotated with the disorder-specific circuit fingerprint that overlies the shared deficit (mood disorders: Qin et al., 2024; Wu et al., 2024; anxiety disorders: Akiki et al., 2025; Dalton et al., 2025; PTSD: Keller et al., 2022; borderline personality disorder: Denny et al., 2024; Sloan et al., 2026; Wong et al., 2026; substance use disorders: Hand et al., 2024; ADHD/OCD: Sloan et al., 2026; Wong et al., 2026).
(C) Layer 3. Four families of individual-difference moderators that act on Layers 1 and 2: developmental history, in which early adversity reaches adult dysregulation via coping, personality, self-efficacy, and attachment (Coelho et al., 2026a, 2026b; Kluwe-Schiavon et al., 2022); dispositional regulatory tendency, which maps onto lateral prefrontal activity and amygdala reactivity (Morawetz & Basten, 2024); regulatory mode, since explicit and implicit regulation differ in sensitivity to prefrontal stimulation (Gao et al., 2024; Qiu et al., 2023); and stimulation parameters, since target, current-flow model, and outcome channel each change the observed effect (Coelho et al., 2025; Gao et al., 2024; McAleer et al., 2023; Meinzer et al., 2024). The diagram is a didactic simplification of a distributed, bidirectional circuit and is not intended as an anatomically exhaustive map. The lateral-view brain illustration used as the anatomical background in Panel (A) was generated with a generative artificial-intelligence image model and curated by the author; all schematic elements (nodes, pathways, labels, and Panels B and C) were drawn programmatically and represent the author’s conceptual synthesis of the cited literature, not empirical data.

## 3. Emotion Regulation as a Transdiagnostic Mechanism: A Disorder-by-Disorder Synthesis

Whether ER genuinely qualifies as a transdiagnostic construct, rather than simply a process that happens to be measured across many disorders, has recently been tested directly and repeatedly using complementary methods and largely non-overlapping datasets. The question has a history. A voxel-based morphometry meta-analysis of 193 studies across six diagnostic groups had already identified a common substrate of gray-matter loss in the dorsal anterior cingulate cortex and bilateral anterior insula (Goodkind et al., 2015), and activation-likelihood meta-analyses of cognitive-control and emotional-processing tasks had shown common patterns of prefrontal hypoactivation and amygdala-hippocampal hyperactivation across psychiatric disorders (McTeague et al., 2017, 2020). In the reappraisal literature specifically, a systematic review of 32 clinical studies identified reduced ventrolateral and dorsolateral prefrontal recruitment as a core deficit shared across diagnoses, with amygdala hyperactivity specific to mood disorders and dorsal cingulate–parietal hypoactivity specific to anxiety disorders (Zilverstand et al., 2017), and a coordinate-based meta-analysis of mood and anxiety disorders found reduced recruitment of the fronto-parietal regulatory network alongside increased insular and cerebellar activation (Picó-Pérez et al., 2017).

What the newest work adds is a direct test of whether ER, as a construct, has a neural signature that is separable from these general disruptions. A large coordinate-based meta-analytic effort reanalyzed data from ten prior meta-analyses spanning current RDoC domains and then conducted an independent meta-analysis contrasting brain activation during ER between patients with psychiatric disorders and healthy controls. The results showed that ER shares neural territory with several RDoC domains, particularly those tied to cognitive and social processing, while retaining a distinct signature centered on the inferior and medial frontal gyrus; critically, dorsomedial prefrontal cortex involvement in ER recurred across diagnostically heterogeneous clinical samples, supporting ER’s status as a genuine transdiagnostic dimension with implications for both diagnostic refinement and treatment design (Morawetz et al., 2025). Complementary behavioral evidence comes from the two 2026 meta-analyses introduced above (Clamor et al., 2026; Sloan et al., 2026). Wong et al. (2026), in a meta-analysis of 35 task-based fMRI studies, identified significant convergence of hyperactivation in the bilateral amygdala and left insula using canonical activation likelihood estimation (ALE). No significant hypoactivation survived the standard cluster-level family-wise error correction. Reduced anterior cingulate cortex and supplementary motor area activity emerged only with exploratory Bayesian thresholding and should therefore be treated as hypothesis-generating rather than established transdiagnostic abnormalities.

Together, these behavioral and neuroimaging syntheses support the transdiagnostic relevance of ER difficulties and suggest partially convergent neural involvement. However, convergence across different measures and analytical approaches does not establish a single invariant neural abnormality across disorders. The following synthesis therefore examines both recurring findings and diagnosis-associated variation.

The evidence synthesized here concerns several related but distinct constructs. Emotional reactivity describes responses to affective stimuli; habitual strategy use concerns how frequently individuals report using particular strategies; self-reported dysregulation concerns perceived difficulties managing emotions; and task-based regulatory performance concerns outcomes under specified experimental instructions. Evidence in one domain does not establish impairment in the others or demonstrate that all reflect a single neural mechanism. Their proposed integration within the LTM-ER requires direct tests of convergence and dissociation across measures. The following synthesis therefore examines both recurring findings and diagnosis-associated variation.

Mood disorders. In major depressive disorder (MDD), a dedicated neuroimaging meta-analysis reported hyperactivity in regions tied to emotional experience (insula, postcentral gyrus) alongside hypoactivity in prefrontal cognitive-control regions during regulation, relative to healthy controls (Wu et al., 2024). A comparative meta-analysis of unaffected first-degree relatives of patients with MDD and bipolar disorder (BD) extends this picture to familial risk, finding that relatives of MDD probands show amygdala and parahippocampal hyperactivation during emotional processing, while relatives of BD probands show a partially distinct pattern of parahippocampal hyperactivation together with striatal and occipital hypoactivation, evidence that even before illness onset, related but non-identical circuit signatures already distinguish these two mood-disorder trajectories (Qin et al., 2024). This distinction matters for the transdiagnostic argument: it shows that a shared vulnerability circuit (prefrontal-limbic dysregulation, broadly construed) can nonetheless carry disorder-specific “fingerprints”, a nuance any transdiagnostic model must accommodate rather than average away.

Anxiety disorders. A comprehensive review of the neural circuit basis of pathological anxiety argues that anxiety disorders arise not only from heightened reactivity within acute-threat (“fear”) circuits, but from abnormalities distributed across circuits governing sustained and distant threat, reward processing, cognitive control, and social processing, circuits that cut across the traditional boundaries separating generalized anxiety, panic, and social anxiety disorder (Akiki et al., 2025). A complementary meta-analytic review of implicit ER specifically in mood and anxiety disorders similarly finds altered engagement of prefrontal-limbic regulatory circuitry when regulation occurs outside conscious awareness, reinforcing that anxiety-related regulatory failure is not confined to explicit, effortful reappraisal but extends to more automatic regulatory processes (Dalton et al., 2025). Notably, the large-scale behavioral meta-analysis discussed above found that reduced cognitive reappraisal was present in essentially every disorder examined except panic disorder, illustrating that even within the broad anxiety-disorder category, the transdiagnostic pattern is not perfectly uniform (Sloan et al., 2026).

Trauma- and stress-related disorders. A direct comparison of MDD and post-traumatic stress disorder (PTSD) found that both groups showed reduced right inferior frontal gyrus engagement during reappraisal relative to controls, alongside diagnosis-specific hyperconnectivity of the salience network in PTSD, a pattern consistent with a shared regulatory deficit overlaid by disorder-specific network alterations (Keller et al., 2022). This dissociation between a shared “core” deficit and a diagnosis-specific network signature recurs throughout this section and is, in my view, the central organizing pattern of transdiagnostic ER neuroscience.

Personality disorders. Borderline personality disorder (BPD) shows the largest case–control effect size of any disorder yet analyzed for ER difficulties, roughly two-and-a-half times the pooled standard deviation separating patients from controls on the Difficulties in Emotion Regulation Scale (Sloan et al., 2026). Encouragingly, this circuitry appears modifiable: a recent trial found that structured training in cognitive reappraisal normalized whole-brain indices of emotion-regulation-related activity in patients with BPD, moving neural response patterns closer to those of unaffected relatives and healthy controls (Denny et al., 2024). This is one of the more direct pieces of evidence available that circuit-level ER abnormalities are not fixed trait markers but are responsive to targeted behavioral intervention, a point directly relevant to the precision-psychiatry argument developed in Section 6.

Substance use and addictive disorders. Emotional dysregulation is increasingly recognized as a primary driver, rather than a downstream consequence, of relapse in substance use disorders, particularly through the withdrawal/negative-affect stage of the addiction cycle, which recruits dysregulated amygdala, insula, anterior cingulate, and medial prefrontal circuitry; critically, the specific pattern of dysregulation differs by substance, with blunted responses to negative affective stimuli in alcohol dependence contrasted with heightened responses in cocaine dependence (Hand et al., 2024). This substance-specific patterning is a further illustration of the “shared core, divergent details” theme running through this section and argues against treating addiction as a purely reward-driven disorder disconnected from the broader transdiagnostic ER literature.

Neurodevelopmental and other conditions. ADHD and obsessive–compulsive disorder (OCD) provide instructive exceptions to otherwise near-universal patterns. The large-scale behavioral meta-analysis found that, unlike in most other disorders, expressive suppression was not elevated in ADHD or OCD relative to controls (Sloan et al., 2026), while the ALE meta-analysis situates ADHD alongside BPD and BD within a shared amygdala-centered hyperactivation profile (Wong et al., 2026). These partial exceptions are a useful corrective against an overly totalizing transdiagnostic narrative: the evidence supports a broadly shared circuit-level vulnerability, not a claim that every disorder shows an identical regulatory profile on every measure.

Table 1 summarizes this disorder-by-disorder evidence alongside representative recent sources.

These findings map naturally onto a broader clinical reconceptualization already underway independent of neuroimaging: a transdiagnostic processual model of emotion dysregulation organized around core triggers and interpersonal styles has recently been proposed to help clinicians formulate emotion dysregulation across eight distinct diagnostic categories using a single, mechanism-based framework rather than disorder-specific heuristics (Blay et al., 2024). A meta-analysis focused specifically on the efficacy of ER strategy implementation across mental disorders reinforces the practical stakes of this reconceptualization: instructed ER produced small but significant improvements in experiential outcomes across clinical populations, with larger effects for cognitive-change strategies delivered after explicit strategy training and using disorder-relevant stimuli, suggesting that transdiagnostic ER interventions are viable but that their efficacy depends on how, and with what training, they are delivered (Reents et al., 2026). Read together, the neuroimaging, large-sample behavioral, and clinical-conceptual studies converge on the same conclusion from different directions: ER dysfunction is not disorder-bound, its circuit-level and behavioral signatures are now among the best-replicated findings in the field, and interventions built around a single diagnostic label are likely to under-serve patients whose regulatory deficits cut across categories.

This is precisely the argument I have advanced regarding executive dysfunction more broadly. Framing executive dysfunction as a transdiagnostic neurocognitive dimension, encompassing both cool processes (inhibitory control, working memory, cognitive flexibility) and hot processes (ER, motivational control), provides a parsimonious account of why the same intervention targets (e.g., prefrontal circuits supporting cognitive control) recur across the treatment literature for ostensibly distinct disorders (Carvalho, 2026). A recent editorial synthesizing eleven contributions on transdiagnostic and dimensional approaches to mental health further underscores that dimensional, neurocognitive, and brain-body mechanisms, rather than categorical diagnoses, are increasingly seen as the appropriate unit of analysis for mechanism-based intervention design, a view directly continuous with the ER-specific evidence reviewed above (Carvalho et al., 2026).

One clinical implication of this convergence deserves emphasis. If dorsomedial and inferior frontal engagement during regulation recurs across diagnostically heterogeneous groups (Keller et al., 2022; Morawetz et al., 2025; Wong et al., 2026; Wu et al., 2024), then screening for ER capacity, rather than, or in addition to, screening for diagnosis-specific symptom checklists, could plausibly serve as an early, transdiagnostic marker of risk. This would be consistent with dimensional approaches already being piloted for other executive processes, and it would allow clinicians to identify patients who might benefit from ER-focused intervention even before their symptom profile crystallizes into a specific categorical diagnosis. The challenge, as the following two sections illustrate, is that regulatory capacity is not a fixed trait to be screened once: it is shaped by developmental history and it responds unevenly to intervention, which means that any transdiagnostic marker of ER must be interpreted alongside these sources of individual variability rather than as a static, one-time readout.

## 4. Early Adversity and Individual Differences in Emotion-Regulatory Circuitry: A Clinical Illustration

A transdiagnostic, mechanism-based account of ER is only useful if it can explain why some individuals develop persistent emotion dysregulation following comparable exposures while others do not. Early-life trauma offers a clear test case. In a Portuguese cohort, we found that the relationship between early-life trauma and adult negative emotional states was not direct but was substantially mediated by emotion-regulation difficulties, coping styles, and personality traits, indicating that early adversity shapes adult affect primarily through its downstream effects on regulatory capacity and dispositional style rather than through a uniform, trauma-type-specific pathway (Coelho et al., 2026a). A related analysis from the same research program showed that self-efficacy and attachment style further mediate this same pathway, suggesting that beliefs about one’s own regulatory competence and relational security constitute additional, partially independent routes through which early adversity is either buffered or amplified into adult emotional dysregulation (Coelho et al., 2026b). Consistent with this, longitudinal work on the psychological impact of the COVID-19 pandemic in Portugal found that neuroticism and difficulties in ER, particularly reliance on emotional suppression rather than cognitive reappraisal, predicted depression, anxiety, and stress symptoms across repeated assessments during a shared, population-level stressor, underscoring that personality and regulatory style, rather than exposure severity alone, differentiate resilient from vulnerable trajectories (Kluwe-Schiavon et al., 2022).

The Portuguese cohort findings concern statistical indirect associations involving ER difficulties, coping, personality, self-efficacy, and attachment; they do not directly demonstrate mediation through neural ER circuitry (Coelho et al., 2026a, 2026b). These psychological findings are compatible with, but do not establish, the possibility that developmental and dispositional characteristics relate to variation in regulatory networks, as suggested by the broader neuroimaging literature on individual differences in ER (Morawetz & Basten, 2024). Cross-sectional mediation analyses cannot establish temporal ordering or causal pathways. Testing the proposed neural extension requires studies that assess adversity, psychological processes, and neural ER measures together, preferably longitudinally.

Sleep represents a further candidate source of person-level and within-person physiological variation relevant to ER. Goldstein and Walker (2014) proposed that sleep, particularly rapid-eye-movement sleep, supports affective homeostasis. In an exploratory cross-sectional study of healthy adults, Federico et al. (2022) associated poorer self-reported sleep quality with differences in resting-state connectivity in limbic and fronto-temporo-parietal networks and greater anxiety and depressive symptoms; these findings do not establish a causal effect on ER. Complementing this observational evidence, Tamm et al. (2019) found that sleep restriction reduced self-rated cognitive-reappraisal success in young adults without detectable corresponding changes in brain activation or connectivity. Together, these studies motivate prospective tests of sleep-related variation in subjective regulatory success and neural measures, without assuming that the two necessarily change together. Within the LTM-ER, habitual sleep quality and recent sleep restriction are therefore candidate person-level and state-related factors whose contributions require direct testing.

## 5. Neuromodulation as a Mechanistic Probe and an Intervention Target

Non-invasive brain stimulation (NIBS), principally transcranial direct current stimulation (tDCS) and transcranial magnetic stimulation (TMS), has been used both to test causal models of ER circuitry and to explore its therapeutic potential. The overall picture emerging from recent meta-analytic and mechanistic work is one of real but conditional efficacy, strongly moderated by the explicit-versus-implicit nature of the regulation being targeted, the specific prefrontal target, and the outcome measure used. A meta-analysis restricted to explicit-versus-implicit ER found that TMS and tDCS applied over the prefrontal cortex reliably improved explicit ER on both subjective and physiological measures but had no detectable effect on implicit ER (Qiu et al., 2023), whereas a separate multichannel tDCS study specifically targeting the VMPFC in individuals with high trait anxiety reported the opposite pattern, namely significant and sustained improvements in implicit reappraisal following focality-optimized VMPFC stimulation (Gao et al., 2024). These findings suggest that the ventral, VMPFC-centered system supporting implicit regulation can be engaged directly through sufficiently targeted stimulation parameters, whereas conventional dorsolateral-target protocols may largely fail to do so. A broader meta-analysis of NIBS effects on the downregulation of negative emotions similarly reported a significant but small overall effect, driven primarily by repetitive TMS rather than tDCS, with tDCS effects further moderated by electrode placement (Zhang et al., 2022). Comparative work in internalizing psychopathologies extended this nuance to stimulation waveform, demonstrating that theta-band transcranial alternating current stimulation (tACS) produced more consistent improvements in ER and psychopathology than conventional tDCS (McAleer et al., 2023).

The work from my research group is broadly consistent with this pattern of conditional, parameter-sensitive efficacy rather than uniform benefit. Active dorsolateral prefrontal cortex (dlPFC) tDCS combined with cognitive reappraisal produced greater reductions in heart rate, an autonomic outcome, than sham stimulation or reappraisal alone, and dlPFC stimulation reduced heart rate even in the absence of explicit reappraisal instructions. In contrast, VMPFC stimulation produced inconsistent effects, and neither stimulation site reliably altered skin conductance or respiratory rate (Coelho et al., 2025). This dissociation across autonomic channels illustrates a broader point: neuromodulation effects on ER are rarely global, and studies relying on a single outcome measure may overestimate or underestimate efficacy. Earlier meta-analytic work from our group examining the P3 event-related potential found that anodal tDCS increased parietal P3 amplitude in a target- and task-dependent manner, specifically following left dlPFC stimulation during oddball tasks and right inferior frontal gyrus stimulation during working-memory tasks (Mendes et al., 2022). These findings reinforce the idea that electrophysiological markers of prefrontal engagement are themselves highly sensitive to stimulation parameters rather than functioning as a uniform dose–response indicator. Not every mechanistic hypothesis in this domain has been confirmed. In a subsequent sham-controlled crossover study, phase-specific alpha tACS applied over the dlPFC failed to enhance cognitive reappraisal or autonomic responses, providing positive evidence for the null hypothesis across physiological, subjective, and affective outcomes (Coelho et al., 2026b). This null finding is informative rather than discouraging because it demonstrates that not all oscillatory or waveform manipulations of prefrontal activity translate into meaningful ER improvements and cautions against assuming that any protocol targeting “the prefrontal cortex” necessarily engages ER-relevant circuitry.

These findings concern different levels of inference. Heart-rate reduction indicates autonomic modulation, but its occurrence without explicit reappraisal limits its interpretation as evidence of ER-specific improvement (Coelho et al., 2025). Likewise, the cited P3 effects were observed during oddball and working-memory tasks and provide evidence relevant to cognitive processing rather than validation of an ER-specific biomarker (Mendes et al., 2022). Physiological modulation should therefore be distinguished from demonstrated engagement of the intended neural target, improved performance on a defined regulatory task, and clinically meaningful benefit. Establishing links between these outcomes requires appropriate control conditions and direct tests; change in one does not establish change in the others.

A recent multi-site position paper on the neural mechanisms of tDCS raises a broader methodological issue that is directly relevant to this literature. Much of the inconsistency observed across NIBS studies may reflect insufficient characterization of individual differences in current flow, cortical excitability, and network state at the time of stimulation, leading the authors to recommend combining stimulation with neuroimaging or electrophysiological measures to verify target engagement directly rather than assuming it (Meinzer et al., 2024). Considered alongside the explicit-versus-implicit dissociation reported by Gao et al. (2024) and Qiu et al. (2023), and the autonomic-marker dissociation reported by Coelho et al. (2025), these findings suggest that NIBS is best conceptualized not as a universally effective ER intervention but as a mechanistic probe whose therapeutic yield depends on matching stimulation parameters to the regulatory process, prefrontal target, and outcome channel under investigation. Section 3′s disorder-by-disorder synthesis introduces an additional stratifying variable that is rarely incorporated into NIBS trial design. Given that borderline personality disorder, addictive disorders, and mood disorders exhibit partially distinct neural signatures despite sharing a common regulatory core (Hand et al., 2024; Qin et al., 2024; Wong et al., 2026), it is plausible that the optimal stimulation target and waveform may also differ across diagnostic groups, rather than a single montage being expected to generalize uniformly across disorders.

Within the ER literature reviewed here, NIBS currently provides a stronger basis for probing regulatory mechanisms than for establishing a transdiagnostic therapeutic intervention. Parameter-dependent effects can inform hypotheses about circuit engagement, particularly when stimulation is combined with neural measurements. However, changes in experimental ER outcomes do not by themselves demonstrate durable improvements in symptoms or everyday functioning. The proposed therapeutic extension requires adequately powered, sham-controlled clinical studies with follow-up and clinically meaningful outcomes.

Taken together, the evidence reviewed in this section suggests a practical principle for the field: NIBS protocols aimed at improving ER should be designed and reported according to at least three explicit specifications. First, studies should indicate whether the intended regulatory process is explicit or implicit, given that these modes differ in their sensitivity to prefrontal stimulation (Gao et al., 2024; Qiu et al., 2023). Second, studies should specify the prefrontal target and current-flow model used, since dorsolateral and ventromedial targets often produce dissociable and sometimes opposing effects (Coelho et al., 2025; Gao et al., 2024). Third, studies should clearly identify the outcome channel used to assess efficacy, whether subjective reports, autonomic indices, or electrophysiological markers, because these measures do not necessarily change in parallel (Coelho et al., 2025; Mendes et al., 2022). Studies that fail to specify these dimensions risk producing findings that are difficult to interpret and compare across laboratories. Similarly, meta-analyses that pool across these dimensions without stratification may obscure precisely the individual- and parameter-level variability that is most relevant for understanding how neuromodulation influences emotion regulation.

## 6. A Layered Transdiagnostic Model of Emotion Regulation (LTM-ER)

The evidence reviewed in Section 2, Section 3, Section 4 and Section 5 can be organized into a single theoretical structure that I will call the Layered Transdiagnostic Model of Emotion Regulation (LTM-ER). Its central claim is that emotion dysregulation across psychiatric conditions is generated by three hierarchically nested layers, each supported by a distinct body of evidence, measured at a different level, and associated with a different clinical implication (Table 2; Figure 1). The model is deliberately parsimonious: it introduces neither new anatomical components nor unmeasured constructs. Rather, it provides an explicit account of how components already identified in the literature relate to one another and generates a set of predictions that can be tested using existing methods.

Layer 1: The Shared Transdiagnostic Core. At the foundation of the model is a regulatory circuit whose disruption recurs across diagnostic categories. This proposed component draws on evidence of cross-diagnostic dmPFC engagement during ER, alongside IFG involvement in regulatory processing (Morawetz et al., 2025). Wong et al. (2026) identified canonical ALE convergence of hyperactivation in the bilateral amygdala and left insula, whereas ACC hypoactivation emerged only through exploratory Bayesian thresholding and did not survive standard cluster-level FWE correction. The ACC remains relevant to the broader conceptual architecture, but its hypoactivation is not treated here as an established transdiagnostic abnormality. These findings are considered alongside evidence from TMS-fMRI studies of reappraisal circuitry (He et al., 2023) and associations between intrinsic connectivity and regulatory success (Morawetz et al., 2024). The behavioral and neural evidence supporting this layer should be distinguished. Large-scale studies provide substantial evidence of elevated self-reported ER difficulties across diagnostic groups (Clamor et al., 2026; Sloan et al., 2026), whereas neuroimaging findings suggest partial convergence that varies with the populations, tasks, contrasts, and analytical methods examined (Morawetz et al., 2025; Wong et al., 2026). Layer 1 therefore represents a convergent model of shared regulatory processes, not an invariant neural circuit or a uniform pattern of dysfunction across all disorders and individuals. The correspondence between this proposed neural core and behavioral ER difficulties remains a hypothesis requiring direct multimodal testing.

Layer 2: Disorder-Specific Circuit Fingerprints. The term “fingerprints” is used descriptively to denote relative patterns of neural divergence associated with diagnostic groups or risk states, superimposed on the proposed shared core. It does not imply that these patterns are unique to a disorder, consistently present in every affected individual, or validated for diagnostic classification. Moreover, findings from familial-risk samples and emotional-processing paradigms provide contextual evidence rather than direct demonstrations of ER abnormalities in diagnosed populations. These include the parahippocampal and striatal-occipital profile associated with bipolar-disorder risk (Qin et al., 2024), salience-network hyperconnectivity in PTSD (Keller et al., 2022), anxiety-related dysregulation extending into reward and social-cognitive systems (Akiki et al., 2025; Dalton et al., 2025), substance-dependent dysregulation patterns in addiction (Hand et al., 2024), and the amygdala-centered profile with relative preservation of suppression observed in ADHD (Sloan et al., 2026; Wong et al., 2026) (Table 1). At the behavioral level, Layer 2 corresponds to disorder-specific gradients in ER facets, such as rumination in depression and non-acceptance in personality disorders (Clamor et al., 2026). The clinical implication is that interventions targeting the shared core may require disorder-specific adaptations to improve disorder-specific outcomes.

Layer 3: Person-Level and Contextual Moderators. The third layer distinguishes person-level characteristics from task and intervention characteristics that may help explain variability in the expression of Layers 1 and 2 and in intervention response. Person-level characteristics include developmental history and psychological factors such as coping, personality, self-efficacy, attachment, and dispositional regulatory tendencies (Coelho et al., 2026a, 2026b; Kluwe-Schiavon et al., 2022; Morawetz & Basten, 2024). Person-level factors may also include time-varying physiological states, such as recent sleep duration or restriction, alongside habitual sleep quality. These should be distinguished from stable dispositions and investigated as candidate sources of within-person and between-person variation, rather than assumed to exert established moderating effects (Goldstein & Walker, 2014; Federico et al., 2022; Tamm et al., 2019). Task and intervention characteristics include explicit versus implicit regulatory mode, stimulation target, waveform, dose, and individual current distribution (Gao et al., 2024; Qiu et al., 2023; McAleer et al., 2023; Meinzer et al., 2024). The outcome channel is a measurement characteristic that influences which effects are detected, rather than a stimulation parameter or person-level trait (Coelho et al., 2025; Mendes et al., 2022). These distinctions allow Layer 3 to accommodate both individual variability and the conditions under which regulation is assessed or modified. The specific moderating roles of these factors within the LTM-ER require direct testing.

The LTM-ER generates at least five predictions that distinguish it from both purely disorder-specific models and undifferentiated accounts of general dysregulation. These predictions differ in their proximity to existing evidence. Findings of cross-diagnostic neural convergence and parameter-dependent stimulation effects provide indirect support for aspects of Predictions 1 and 4. However, they do not directly establish severity scaling independently of diagnosis or the superiority of prospectively matched stimulation protocols. Prediction 2 extends observed group differences to out-of-sample diagnostic classification after accounting for shared variance; Prediction 3 extends psychological mediation findings to neural outcomes; and Prediction 5 proposes differential transfer of intervention effects. These extensions remain prospective hypotheses. None of the five predictions should be interpreted as already validated in its full form.

Prediction 1 (Cross-Diagnostic Convergence and Severity Scaling): when assessed using comparable regulatory tasks and measures, a reproducible neural component shared across diagnostic groups will explain variation in ER difficulties or regulatory performance beyond diagnostic category. Associations with self-reported difficulties and task-based performance should be tested separately. The component’s anatomical composition and generalizability require independent validation rather than being fixed in advance as an invariant regional imbalance. Prediction 2 (Fingerprint Specificity): after statistically removing the shared Layer-1 component, residual circuit patterns will continue to classify diagnostic category above chance. Prediction 3 (Developmental Pathways): in longitudinal data, coping, personality, self-efficacy, and attachment are candidate mediators of associations between early adversity and subsequent variation in the proposed Layer-1 neural component. Testing this prediction requires temporally ordered assessments and attention to baseline differences and confounding; the existing cross-sectional psychological findings do not establish this neural pathway. A distinct moderation hypothesis would ask whether a person-level characteristic changes the strength of the adversity–neural association and would require an interaction test. Prediction 4 (Parameter-Conditional Intervention Effects): neuromodulation protocols with stimulation parameters prospectively matched to the intended regulatory process will produce larger and more consistent ER effects than comparison protocols without such matching. Outcome measures should be selected a priori to test the hypothesized effects on subjective, autonomic, or neural responses, rather than treated as stimulation parameters (Coelho et al., 2025; Gao et al., 2024; Qiu et al., 2023). Prediction 5 (Differential Transfer): interventions engaging shared regulatory processes are predicted to improve ER across diagnostic groups, while Layer-2-informed adaptations may provide additional benefits for disorder-relevant symptoms.

Operationalizing and testing the layered structure. One possible approach is to use participant-level data from multiple diagnostic groups and controls completing matched ER paradigms. A preregistered multigroup latent-variable or hierarchical model could estimate a neural component with loadings shared across groups alongside diagnosis-associated deviations. A shared mean difference from controls should be distinguished from shared neural–behavioral covariance, and any constraints used to separate the components should be stated explicitly. The layered model should be compared with shared-only and diagnosis-specific alternatives using held-out predictive performance and component stability. A decomposition imposed by the analysis is not, by itself, evidence that the proposed layers exist.

Prediction 2 would require testing whether diagnosis-associated components provide incremental diagnostic information beyond the shared component and clinical covariates. Feature selection, component estimation, harmonization, and confound adjustment should be fitted within training data, with model tuning confined to internal validation and final evaluation performed in an independent cohort, preferably including held-out sites. Analyses should account for ER and broader symptom severity, comorbidity, medication exposure, and site effects through prespecified covariate models and sensitivity analyses. Classification should be evaluated using permutation-based chance estimates, balanced performance metrics, and uncertainty intervals, including comparisons with models using clinical covariates alone.

Evidence against the proposed separation would include failure to recover a reproducible shared component, failure of that component to relate to prespecified ER outcomes across groups, or absence of incremental diagnostic information in the residual patterns under adequately powered and reliable measurement conditions. If the layered model does not outperform simpler alternatives in independent data, its additional complexity would not be supported. These outcomes would challenge the operationalized model rather than justify redefining the layers after observing the results.

Relation to Existing Frameworks. The LTM-ER is not proposed in isolation and should be considered in relation to four major theoretical traditions. First, the process model of emotion regulation and its extensions (Gross, 2015; McRae & Gross, 2020) conceptualize regulation as a sequence of stages involving situation selection, situation modification, attentional deployment, cognitive change, and response modulation. Whereas these models specify what regulation is, the LTM-ER focuses on how regulatory systems fail across psychiatric conditions. Layer 2, for instance, maps naturally onto disorder-specific disruptions in regulatory stages, consistent with facet-specific findings reported by Clamor et al. (2026), while Layer 3 incorporates the distinction between deliberate and automatic regulation (Dalton et al., 2025; Gao et al., 2024; Qiu et al., 2023).

Second, the RDoC framework (Cuthbert, 2022) situates ER-related processes within the Negative Valence and Cognitive Systems domains and emphasizes multilevel measurement from neural circuits to self-report. The coordinate-based meta-analysis reviewed earlier (Morawetz et al., 2025) effectively constitutes an RDoC-style test of the distinctiveness of ER. The LTM-ER extends this perspective by distinguishing between domain-general and diagnosis-linked aspects of regulatory circuitry.

Third, the Hierarchical Taxonomy of Psychopathology (HiTOP) (Kotov et al., 2021) conceptualizes psychopathology as a hierarchy comprising a general factor, broad spectra, and narrower syndromes. The layered structure of the LTM-ER offers a plausible neural analogue, with Layer 1 corresponding to the general factor and Layer 2 corresponding to spectra and syndromes. Recent efforts to integrate RDoC and HiTOP (Michelini et al., 2021) explicitly call for this type of bridge between dimensional psychopathology and neural mechanisms.

Fourth, the model builds directly upon the first generation of transdiagnostic neuroimaging syntheses (Goodkind et al., 2015; McTeague et al., 2017, 2020; Picó-Pérez et al., 2017; Zilverstand et al., 2017) and the canonical neural model of reappraisal (Buhle et al., 2014; Etkin et al., 2015; Ochsner et al., 2012). What differentiates the LTM-ER from these previous accounts is its explicit treatment of the relationship between shared and disorder-specific circuitry as a layered, falsifiable hypothesis; its reliance on causal neuromodulation evidence rather than purely correlational imaging findings; and its integration of developmental history and dispositional regulatory tendencies as core moderators rather than nuisance variables.

## 7. Toward Precision Psychiatry: Integrating Circuits, Disorders, Traits, and Biomarkers

If the three layers of the LTM-ER are broadly correct, that is, if ER is a transdiagnostic mechanism shaped jointly by circuit-level properties (Azarias et al., 2025; He et al., 2023; Morawetz & Basten, 2024; Morawetz et al., 2024, 2025), disorder-specific circuit fingerprints superimposed on a shared regulatory core (Denny et al., 2024; Hand et al., 2024; Keller et al., 2022; Qin et al., 2024; Wong et al., 2026), early-adversity-related individual differences (Coelho et al., 2026a, 2026b, 2026c; Kluwe-Schiavon et al., 2022), and stimulation-parameter-dependent malleability (Coelho et al., 2025, 2026b; Gao et al., 2024; McAleer et al., 2023; Meinzer et al., 2024; Mendes et al., 2022; Qiu et al., 2023; Zhang et al., 2022), the natural next step is integration: combining these levels of description into biomarker-informed, mechanism-based interventions rather than treating each as a separate literature. 

This ambition mirrors a broader movement in psychiatry toward precision approaches. A recent critical appraisal of biomarkers for affective disorders argues that disrupted brain-body communication may be best captured not by any single biomarker but by clusters of interconnected peripheral and central markers that together constitute a disease “biosignature,” better reflecting the multifactorial pathophysiology of mood disorders than any single measure could (Berry et al., 2025). A comprehensive review of candidate psychiatric biomarkers across diagnostic categories reaches a more cautious conclusion, noting that despite decades of investment, very few neuroimaging, electrophysiological, or other candidate markers have yet been validated to the point of clinical actionability and recommending that the field focus resources on a limited number of the most promising candidates for definitive testing rather than continuing to generate isolated biomarkers (Abi-Dargham et al., 2023). The autonomic and electrophysiological measures discussed in Section 5 may be useful exploratory outcomes, but their sensitivity to stimulation does not establish ER specificity or suitability for patient stratification. Their use as engagement or response biomarkers would require evidence of reliability, a defined relationship with the intended regulatory process or neural target, and incremental predictive value in independent samples.

Precision medicine frameworks developed outside psychiatry offer a template for how such integration might proceed. Multi-omic and multimodal biomarker panels are already being used elsewhere in medicine to stratify patients, monitor treatment response, and reduce exposure to ineffective interventions, and similar approaches have been proposed for mental healthcare through the integration of genetic, neuroimaging, and behavioral data (Scala et al., 2023). Applied to ER, this would mean treating regulatory capacity not as a single trait assessed once, but as a multidimensional profile spanning circuit connectivity, disorder-relevant circuit fingerprints, autonomic reactivity, personality and coping style, and developmental history. Such a profile could be used prospectively to identify patients most likely to benefit from cognitive-behavioral reappraisal training, NIBS targeting a specific prefrontal site, or interventions focused on coping and attachment-related vulnerabilities identified in early-adversity research (Coelho et al., 2026a, 2026b). Realizing this integration will also require the methodological broadening advocated at the outset of this Perspective, including multimodal, longitudinal, and ecologically valid designs capable of linking laboratory-based regulatory profiles to regulation in daily life (Petrova & Gross, 2023).

A stratified ER research pipeline might proceed in three stages. First, at intake, patients would be characterized on the developmental and dispositional dimensions already shown to moderate the pathway from adversity to dysregulation, including coping style, personality, self-efficacy, and attachment (Coelho et al., 2026a, 2026b; Kluwe-Schiavon et al., 2022), alongside a brief laboratory-based or ecologically sampled measure of regulatory strategy use and, where clinically relevant, the disorder-specific circuit fingerprints summarized in Table 1. Second, for patients being considered for neuromodulation, a brief individual-differences localization procedure, for example structural imaging to inform current-flow modeling or an explicit-versus-implicit regulation assessment, could help determine whether a dorsolateral or ventromedial target and which stimulation waveform are mechanistically appropriate rather than defaulting to a single standard montage (Coelho et al., 2025; Gao et al., 2024; McAleer et al., 2023). Third, outcome monitoring would incorporate multiple channels simultaneously, including subjective symptom reports, autonomic indices such as heart rate and skin conductance, and, where feasible, electrophysiological markers such as P3 amplitude, thereby reducing the risk that partial changes in one domain are interpreted as global success or failure (Coelho et al., 2025; Mendes et al., 2022). None of these three stages requires technology beyond what is already employed in the individual studies reviewed here; what remains lacking is their systematic integration within a single patient pathway. Building such a pathway is, in my view, a realistic near-term objective for transdiagnostic ER research rather than a distant aspiration.

## 8. Practical Recommendations and Future Directions

The value of a Perspective lies in what it changes about how work is done. This section therefore translates the LTM-ER into specific recommendations for three audiences—researchers designing ER studies, investigators running neuromodulation trials, and clinicians assessing and treating emotion dysregulation—followed by the open questions I consider most urgent (Table 3).

### 8.1. For Emotion-Regulation Research Design

First, sample and analyze across diagnostic categories by default. Studies restricted to a single diagnosis cannot, in principle, separate Layer 1 from Layer 2, and the field already has the behavioral evidence needed to justify transdiagnostic recruitment (Clamor et al., 2026; Sloan et al., 2026). A practical minimum is two clinical groups plus controls, with ER difficulty measured continuously so that Prediction 1 (severity scaling) can be tested directly. Second, report the shared and the disorder-specific components separately. Analyses should first estimate the circuit pattern common to all clinical groups and then test whether residual patterns distinguish diagnoses (Prediction 2), rather than reporting only a single patients-versus-controls contrast (Morawetz et al., 2025; Wong et al., 2026). Third, measure Layer-3 moderators in every study, not only in studies about them. Early adversity, coping style, personality, self-efficacy, and attachment can be assessed in under thirty minutes with validated instruments and have already been shown to mediate the adversity-to-dysregulation pathway (Coelho et al., 2026a, 2026b; Kluwe-Schiavon et al., 2022); omitting them discards the variable most likely to explain between-participant heterogeneity in circuit findings (Morawetz & Basten, 2024). Fourth, follow the methodological broadening already called for in the field: multimodal designs, repeated measurement, and ecological sampling of regulation in daily life, so that laboratory circuit measures can be related to real-world regulatory behavior (Petrova & Gross, 2023).

### 8.2. For Neuromodulation Trials

The three-specification principle stated in Section 5 should be treated as a minimum reporting standard: every NIBS-ER study should state (i) whether explicit or implicit regulation is targeted, (ii) which prefrontal target and current-flow model is used, and (iii) which outcome channels are measured (Coelho et al., 2025; Gao et al., 2024; Mendes et al., 2022; Qiu et al., 2023). Beyond reporting, three design recommendations follow from the model. First, match target to regulatory mode a priori rather than testing a single montage: the available evidence favors dorsolateral targets for explicit reappraisal and autonomic outcomes (Coelho et al., 2025; Qiu et al., 2023) and ventromedial targets for implicit regulation in high-anxiety samples (Gao et al., 2024), and trials should be powered to detect this interaction. Second, individualize current flow. Structural imaging-based current-flow modeling is now routine in tDCS research, and the multi-site position paper on tDCS mechanisms makes clear that unmodeled inter-individual variability in current distribution is a leading candidate explanation for inconsistent effects (McAleer et al., 2023; Meinzer et al., 2024). Third, verify target engagement with concurrent neuroimaging or electrophysiology wherever feasible, so that a null behavioral result can be attributed either to the hypothesis or to a failure of engagement (He et al., 2023; Meinzer et al., 2024). Trials designed this way test Prediction 4 directly and, if the model is correct, should show systematically larger effects than the pooled estimates currently available.

### 8.3. For Clinical Assessment and Intervention

The clinical implications of the LTM-ER are model-derived proposals requiring prospective validation, rather than established standards of care. The model motivates evaluating whether assessment of ER difficulties adds clinically useful information beyond routine diagnostic and symptom assessment, and whether developmental and dispositional characteristics improve treatment selection. It also motivates testing interventions intended to engage shared regulatory processes, with disorder-relevant adaptations where appropriate. However, the model does not establish that such interventions should be prioritized as first-line treatments across diagnoses.

Four priorities for clinical evaluation follow from this framework. First, prospective studies could examine whether brief, validated measures of ER difficulties add useful information to routine assessment across mood, anxiety, trauma-related, personality, and substance-related presentations, building on evidence of cross-diagnostic ER difficulties (Clamor et al., 2026; Sloan et al., 2026). Second, studies could test whether developmental history, coping, personality, self-efficacy, and attachment improve the prediction of treatment response beyond baseline symptoms and ER difficulties; the psychological associations reviewed here motivate this possibility but do not establish a treatment-selection rule (Coelho et al., 2026a, 2026b). Third, comparative trials could evaluate whether interventions intended to engage shared regulatory processes, such as reappraisal training, produce benefits across diagnoses and whether disorder-relevant adaptations provide additional benefit, for example by addressing non-acceptance or rumination where these difficulties are prominent (Clamor et al., 2026; Denny et al., 2024; Reents et al., 2026). Fourth, research could assess whether combining subjective reports with autonomic and cognitive-control measures improves treatment monitoring sufficiently to justify the additional assessment burden, given that these outcomes may change differently following intervention (Coelho et al., 2025; Mendes et al., 2022). These proposals are conceptually consistent with a processual, formulation-based approach to emotion dysregulation (Blay et al., 2024) and with the research pipeline outlined in Section 7, but their incremental clinical utility requires direct evaluation.

### 8.4. Priority Open Questions

Five questions follow directly from the model and, in my view, deserve priority. (1) Is the Layer-1 signature stable within individuals over time and across regulatory strategies, or is it state-dependent? Longitudinal task-based imaging in transdiagnostic samples is required to answer this question. (2) Do Layer-2 fingerprints predict differential treatment response, or are they epiphenomena of symptom expression? Prospective trials stratified according to circuit fingerprints would provide a direct test of Prediction 5. (3) At what stage of development do Layer-3 moderators begin to shape Layer-1 circuitry, and is this developmental window modifiable? Cohort studies linking early adversity, dispositional variables, and adolescent brain development represent the most appropriate design for addressing this issue. (4) Which autonomic and electrophysiological markers are sufficiently reliable at the individual level to serve as engagement or response biomarkers in routine clinical care (Abi-Dargham et al., 2023; Berry et al., 2025)? (5) Can a combined Layer-1/Layer-3 profile obtained at intake predict which patients are most likely to benefit from behavioral reappraisal training, targeted neuromodulation, or interventions aimed at dispositional vulnerabilities, thereby fulfilling the central promise of precision psychiatry applied to ER (Scala et al., 2023)?

## 9. Conclusions

Emotion regulation is now among the best-replicated transdiagnostic mechanisms in clinical psychological science: independent meta-analyses spanning fifteen diagnostic categories, hundreds of studies, and tens of thousands of participants converge on large, significant ER impairment across mood, anxiety, trauma-related, personality, and addictive disorders, with a partially dissociable but convergent neural signature across prefrontal-limbic circuits and large-scale networks. The novel contribution of this Perspective is not to re-demonstrate that convergence, but to organize and formalize it. The Layered Transdiagnostic Model of Emotion Regulation proposed here holds that a shared regulatory core (Layer 1) is consistently overlaid by disorder-specific circuit fingerprints (Layer 2) and modulated by individual-difference moderators (Layer 3), and it yields five testable predictions and a set of concrete recommendations for research, neuromodulation trials, and clinical practice. The disorder-by-disorder synthesis shows that the shared regulatory core is consistently overlaid by disorder-specific circuit fingerprints—distinct in bipolar disorder relative to major depression, in borderline personality disorder relative to other conditions, and in substance-specific patterns within addiction—and a growing body of causal evidence, chiefly from combined TMS/tDCS-fMRI and stimulation studies, clarifies how the shared and divergent components of this circuitry can be probed and, under the right conditions, modulated. At the same time, the clinical and stimulation studies reviewed here converge on a common caveat: neither vulnerability to emotion dysregulation nor responsiveness to intervention is uniform across individuals or across diagnoses.

Early-adversity research demonstrates that personality, coping style, self-efficacy, and attachment jointly determine whether early trauma translates into adult emotional dysregulation, while the neuromodulation literature demonstrates that the same stimulation target can produce divergent effects depending on whether regulation is explicit or implicit, which outcome channel is measured, how precisely current flow is characterized, and plausibly which disorder-specific circuit fingerprint a given patient carries. Taken together, these findings argue for a shift away from disorder-specific, one-size-fits-all models of emotion regulation and its treatment, toward mechanism-based, individually stratified approaches that integrate circuit-level markers, disorder-specific fingerprints, autonomic and electrophysiological indices, and developmental and dispositional history. Building this integration—rather than continuing to generate isolated findings within any one of these levels—is, in my view, the most productive direction for the next generation of emotion-regulation neuroscience.

## Figures and Tables

**Table 1:** Disorder-specific neural and behavioral emotion-regulation findings overlaid on the shared transdiagnostic core (Layer 2 of the LTM-ER), with representative recent sources.

Diagnostic Category	Representative Neural Finding	Representative Behavioral/ER Finding	Key Recent Sources
Major Depressive Disorder	Hyperactivity in the insula and postcentral gyrus; hypoactivity in prefrontal cognitive-control regions during reappraisal	Elevated rumination; reduced cognitive reappraisal	Clamor et al. (2026); Wu et al. (2024)
Bipolar Disorder	Parahippocampal hyperactivation with striatal and occipital hypoactivation in at-risk relatives	Smallest, but still large, DERS effect size (*g* = 0.94) among disorders analyzed	Qin et al. (2024); Sloan et al. (2026)
Anxiety Disorders (GAD, panic, social anxiety)	Circuit disruption extending beyond fear circuits to sustained-threat, reward, cognitive-control, and social-processing systems	Reduced reappraisal in all anxiety subtypes except panic disorder; altered implicit ER	Akiki et al. (2025); Dalton et al. (2025); Sloan et al. (2026)
Post-Traumatic Stress Disorder	Reduced right inferior frontal gyrus engagement during reappraisal; salience-network hyperconnectivity	Shared regulatory deficit with MDD plus disorder-specific network alteration	Keller et al. (2022)
Borderline Personality Disorder	Amygdala/insula hyperactivation in a transdiagnostic synthesis including BPD; ACC hypoactivation exploratory only; reappraisal training shifted whole-brain ER-related patterns toward comparison groups	Largest DERS case–control effect size of any disorder studied (*g* = 2.55)	Denny et al. (2024); Sloan et al. (2026); Wong et al. (2026)
Substance Use Disorders	Dysregulated amygdala, insula, ACC, and medial prefrontal circuitry; patterning differs by substance	Emotional dysregulation as a primary driver of relapse rather than a downstream symptom	Hand et al. (2024)
ADHD/OCD	Amygdala-centered hyperactivation profile shared with BPD and BD (ADHD)	Suppression not elevated relative to controls (exception to the general transdiagnostic pattern)	Sloan et al. (2026); Wong et al. (2026)

**Table 2:** The three layers of the Layered Transdiagnostic Model of Emotion Regulation (LTM-ER): content, evidence base, measurement level, and central prediction.

Layer	Content	Key Evidence	Measurement Level	Central Prediction
Layer 1: Shared Transdiagnostic Core	Cross-diagnostic dmPFC engagement; IFG involvement in regulatory processing; canonical ALE hyperactivation in bilateral amygdala and left insula; exploratory ACC hypoactivation; proposed regulatory interactions within distributed networks	Azarias et al. (2025); Clamor et al. (2026); He et al. (2023); Morawetz et al. (2024, 2025); Sloan et al. (2026); Wong et al. (2026)	Task-based fMRI during ER; resting-state connectivity; DERS	A reproducible shared neural component explains ER variation beyond diagnosis (P1)
Layer 2: Disorder-Specific Circuit Fingerprints	Patterns overlaid on the core that distinguish diagnostic groups (e.g., parahippocampal/striatal-occipital in BD risk; salience-network hyperconnectivity in PTSD; substance-dependent patterning in SUD)	Akiki et al. (2025); Clamor et al. (2026); Dalton et al. (2025); Denny et al. (2024); Hand et al. (2024); Keller et al. (2022); Qin et al. (2024); Wong et al. (2026); Wu et al. (2024)	Group contrasts after removing the shared component; ER-facet profiles	Residual patterns classify diagnosis above chance (P2); Layer-2-informed adaptations may provide additional benefits for disorder-relevant symptoms (P5)
Layer 3: Person-Level and Contextual Moderators	Developmental history (early adversity via coping, personality, self-efficacy, attachment); dispositional regulatory tendency; explicit vs. implicit regulatory mode; stimulation target, current flow, and outcome channel	Coelho et al. (2025, 2026a, 2026b); Gao et al. (2024); Kluwe-Schiavon et al. (2022); McAleer et al. (2023); Meinzer et al. (2024); Mendes et al. (2022); Morawetz and Basten (2024); Qiu et al. (2023)	Self-report and interview; individual current-flow modeling; multi-channel outcome monitoring	Psychological factors may mediate associations between early adversity and Layer-1 dysfunction (P3); task and intervention characteristics may condition intervention response (P4)

**Table 3:** Research recommendations and proposals for clinical evaluation derived from the LTM-ER, with related model predictions and sources informing their rationale.

Audience	Recommendation	Model Prediction Addressed	Key Sources
Researchers	Recruit at least two clinical groups plus controls; measure ER difficulty continuously	P1 (severity scaling)	Clamor et al. (2026); Morawetz et al. (2025); Sloan et al. (2026)
Researchers	Estimate the shared circuit component first, then test residual disorder-specific patterns	P2 (fingerprint specificity)	Morawetz et al. (2025); Wong et al. (2026)
Researchers	Assess early adversity, coping, personality, self-efficacy, and attachment in every ER study	P3 (developmental pathways)	Coelho et al. (2026a, 2026b); Kluwe-Schiavon et al. (2022); Morawetz and Basten (2024)
Researchers	Use multimodal, repeated, and ecologically sampled designs	Links laboratory circuits to daily life regulation	Petrova and Gross (2023)
NIBS Investigators	Report regulatory mode, target/current-flow model, and outcome channels as a minimum standard	P4 (parameter-conditional effects)	Coelho et al. (2025); Gao et al. (2024); Mendes et al. (2022); Qiu et al. (2023)
NIBS Investigators	Match target to regulatory mode a priori; power for the target × mode interaction	P4	Coelho et al. (2025); Gao et al. (2024); Qiu et al. (2023)
NIBS Investigators	Individualize current flow and verify target engagement with concurrent imaging/EEG	P4	He et al. (2023); McAleer et al. (2023); Meinzer et al. (2024)
Clinicians	Evaluate the incremental clinical utility of transdiagnostic ER assessment at intake	P1	Clamor et al. (2026); Sloan et al. (2026)
Clinicians	Test whether developmental and dispositional characteristics improve treatment selection	P3	Coelho et al. (2026a, 2026b)
Clinicians	Compare interventions targeting shared regulatory processes with approaches incorporating disorder-relevant adaptations	P5 (differential transfer)	Blay et al. (2024); Clamor et al. (2026); Denny et al. (2024); Reents et al. (2026)
Clinicians	Evaluate the added value and feasibility of multimodal outcome monitoring	P4	Coelho et al. (2025); Mendes et al. (2022)

Note. Clinical proposals require prospective validation and do not constitute established standards of care. The cited sources inform the rationale for the recommendations; they do not necessarily provide direct tests of the corresponding model predictions or establish the clinical utility of the proposed applications.

## Data Availability

No new data were created or analyzed in this study. Data sharing is not applicable to this article.

## Abbreviations

The following abbreviations are used in this manuscript:

ACC	Anterior Cingulate Cortex
ADHD	Attention-Deficit/Hyperactivity Disorder
ALE	Activation Likelihood Estimation
BD	Bipolar Disorder
BPD	Borderline Personality Disorder
CIPsi	Psychology Research Centre (Centro de Investigação em Psicologia)
DMN	Default Mode Network
DERS	Difficulties in Emotion Regulation Scale
dlPFC	Dorsolateral Prefrontal Cortex
EEG	Electroencephalography
ER	Emotion Regulation
fMRI	Functional Magnetic Resonance Imaging
GAD	Generalized Anxiety Disorder
HiTOP	Hierarchical Taxonomy of Psychopathology
IFG	Inferior Frontal Gyrus
LTM-ER	Layered Transdiagnostic Model of Emotion Regulation
MDD	Major Depressive Disorder
NIBS	Non-Invasive Brain Stimulation
OCD	Obsessive–Compulsive Disorder
PFC	Prefrontal Cortex
PTSD	Post-Traumatic Stress Disorder
RDoC	Research Domain Criteria
SUD	Substance Use Disorder
tACS	Transcranial Alternating Current Stimulation
tDCS	Transcranial Direct Current Stimulation
TMS	Transcranial Magnetic Stimulation
VLPFC	Ventrolateral Prefrontal Cortex
VMPFC	Ventromedial Prefrontal Cortex
